# Preliminary insight into horse owners’ perceptions of, and attitudes towards, exotic diseases in the United Kingdom

**DOI:** 10.1186/s12917-019-2120-5

**Published:** 2019-10-12

**Authors:** Kelsey L. Spence, Jacqueline M. Cardwell, Josh Slater, Sarah M. Rosanowski

**Affiliations:** 1 Department of Pathobiology and Population Sciences, Royal Veterinary College, Hawkshead Lane, Hatfield, Hertfordshire, AL9 7TA UK; 2 Department of Clinical Science and Services, Royal Veterinary College, Hatfield, Hertfordshire, UK; 3 Centre for Applied One Health Research and Policy Advice, Jockey Club College of Veterinary Medicine and Life Sciences, City University of Hong Kong, Kowloon, Hong Kong; 4 Present Address: Department of Veterinary Clinical Sciences, Faculty of Veterinary and Agricultural Sciences, The University of Melbourne, Werribee, Victoria Australia

**Keywords:** Biosecurity, Equine, Exotic and emerging diseases, Disease preparedness, Risk perceptions

## Abstract

**Background:**

The potential for an exotic disease incursion is a significant concern for the United Kingdom (UK) equine industry. Horse owners’ perceptions of, and attitudes towards, exotic diseases can influence decisions to adopt disease preparedness strategies. The objectives of this study were to describe horse owners’ 1) perceptions of the term ‘exotic disease’, and 2) attitudes towards their risk of being affected by an exotic disease. In order to address these objectives, qualitative content analysis was undertaken on data collected using two open-ended survey questions.

**Results:**

Horse owners (*n* = 423) perceived exotic diseases as 1) belonging somewhere else, and 2) a dangerous threat to their horse(s). The term ‘exotic’ was associated with being foreign, non-native, and out-of-place in the UK. Attitudes towards exotic disease risk were summarised into four categories: 1) responsible horse owners prevent disease, 2) horse owners need support to stop disease spread, 3) risk depends on proximity to the ‘risky’ horse, and 4) some risk is inevitable. A ‘responsible’ owner was aware of health hazards and took actions to protect their horse from disease. Reliance on others, including stakeholders, to uphold disease prevention in the community led to feeling vulnerable to disease threats. When evaluating risk, horse owners considered which horses were the ‘riskiest’ to their horse’s health (horses that travelled, participated in competitions, or were simply unfamiliar) and avoided situations where they could interact. Despite undertaking disease prevention measures, the perceived uncontrollable nature of exotic diseases led some owners to feel an incursion was inevitable.

**Conclusions:**

Without accounting for horse owners’ perceptions of, and attitudes towards, exotic diseases, recommendations to increase preparedness may be ineffective. Improved communication among stakeholders in the industry may assist in clarifying expectations for exotic disease-specific prevention measures. A collaborative approach among horse owners and stakeholders is recommended to improve disease preparedness within the industry.

## Background

Incursions of exotic equine diseases into the United Kingdom (UK) horse industry have been infrequent, but the potential for a future outbreak invokes significant economic and welfare concerns. Exotic equine diseases, defined as those not normally present in the UK, include diseases caused by pathogens such as West Nile virus (WNV), African horse sickness virus (AHS), and equine infectious anaemia (EIA). The increasingly international nature of the horse industry, as well as the changing distribution of vectors capable of transmitting exotic pathogens [1, 2], have contributed to an increased focus on exotic disease preparedness within the industry. Each country in the UK produces their own exotic disease contingency plan that outlines procedures to prepare for, and respond to, an outbreak. Actions recommended in the event of an exotic disease incursion depend on the specific pathogen in question and may also involve additional transnational control strategies (e.g. the AHS control strategy for Great Britain) [3]. Procedures within the contingency plan outline horse owners’ responsibility to follow good biosecurity at all times and remain vigilant for any suspicious clinical signs [4]. If anyone suspects an exotic disease, they must immediately report their suspicions to the government. However, a recent study found that less than 17% of surveyed horse owners in the UK could identify the names of exotic diseases or specific clinical signs associated with them [5], highlighting the potential for delayed or missed detection.

Alongside preparing for an effective exotic disease response, the contingency plan outlines that horse owners should implement disease prevention measures as part of their routine horse management. Several recommended disease prevention measures are similar for both endemic and exotic diseases and include vaccination, quarantine of new arrivals, and good hygiene (e.g. disinfection of items and surfaces) [6]. While disease prevention is highly regulated by governing bodies in the horse racing industry, the non-racing industry is less regulated and therefore might differ in its uptake of recommended disease prevention measures. Despite the differences in regulations, non-racing horses are still at risk of exotic diseases due to the density of the population and international movements to participate in equestrian activities [7, 8]. Non-racing horse owners participate in a wide range of equestrian activities which can increase their likelihood of pathogen exposure [9, 10]. Furthermore, non-racing horse owners may be less likely to implement biosecurity protocols on their yards [11]. The lack of information on whether or not non-racing horse owners are implementing biosecurity measures introduces challenges in evaluating the current level of exotic disease preparedness in the UK.

The uptake of management practices to prevent pathogen introduction and spread depends on a variety of factors, including demographics, problem awareness, perceived responsibility, previously held beliefs, and sociocultural norms [12–18]. Individuals make judgements about the ‘riskiness’ of health hazards and the subsequent level of prevention they should implement to counteract these risks [19]. Horse owners’ attitudes and perceptions towards risk factors of exotic diseases can influence their uptake of disease prevention measures [20]. Without accounting for horse owners’ understanding of the risk of exotic diseases, recommendations to increase preparedness efforts may be ineffective.

The objectives of this study were to describe horse owners’ 1) perceptions of the term ‘exotic disease’, and 2) attitudes towards their risk of being affected by an exotic disease. In order to address these objectives, we conducted qualitative content analysis on data from two open-ended questions on an online questionnaire. A qualitative approach allowed for obtaining insight into the meanings horse owners attributed to exotic diseases and their associated risks.

## Results

### Participant characteristics

A total of 532 responses to the questionnaire were received, of which 423 (79.5%) were included in this study. The remaining 109 responses were excluded from analysis for the following reasons: 19 participants were lost to follow-up after the initial consent question, 5 participants did not meet the inclusion criteria of owning or caring for horses within the UK, and 85 participants did not answer either open-ended questions used for the analysis. Included and excluded participants were similar among several demographic characteristics including age (*p* = 0.43), gender (*p* = 1.00), education (*p* = 0.51), professional role (*p* = 0.92), and horse use (*p* = 0.42). However, a higher proportion of excluded responses were from individuals based in Scotland (*p* = 0.04) and had ten or fewer years of horse experience (*p* < 0.001).

Eighty-eight percent of participants (*n* = 371) owned horses, while 12% (*n* = 51) were equine professionals (e.g. veterinary surgeons, trainers, and grooms) (Table 1). Eighty-nine percent of participants (*n* = 375) had greater than ten years of experience working with horses. Participants reported owning or providing care for a median of 2 horses (IQR 1–4). Most participants (67%, *n* = 285) kept their horse(s) for leisure activities, such as pleasure riding or companionship (herein referred to as ‘leisure’ owners or professionals), while 27% of participants (*n* = 116) kept their horse(s) for competitive activities, such as dressage, eventing or showing (herein referred to as ‘competition’ owners or professionals).

**Table 1:** Demographic characteristics of participants included in the study (*n* = 423)

Variable	Category	Count (%)
Age	18–34	120 (28.4)
	35–54	171 (40.4)
	> 54	103 (24.3)
	No response	29 (6.86)
Gender	Female	389 (92.0)
	Male	8 (1.89)
	No response	26 (6.15)
Education	O-levels	52 (12.3)
	A-levels	122 (28.8)
	Undergraduate degree	136 (32.2)
	Postgraduate degree	78 (18.4)
	No response	35 (8.27)
Role	Horse owner	371 (87.7)
	Horse professional	51 (12.1)
	No response	1 (0.236)
Length of horse experience	≤ 10 years	44 (10.4)
	> 10 years	375 (88.7)
	No response	4 (0.946)
Main horse activity	Leisure	285 (67.4)
	Competition	116 (27.4)
	Other	22 (5.20)
Location	England	350 (82.7)
	Scotland	37 (8.74)
	Wales	20 (4.73)
	Northern Ireland	7 (1.65)
	No response	9 (2.13)

### Perceptions of exotic diseases

Ninety-eight percent of participants (*n* = 415) provided a response about their understanding of the term ‘exotic disease’. Two categories were identified among the responses: 1) exotic diseases belong somewhere else, and 2) exotic diseases are dangerous.

#### Exotic diseases belong somewhere else

Exotic diseases were framed as those which were not natively ‘ours’, and therefore viewed as out-of-place in the UK. Participants described exotic diseases as something which originated elsewhere and could be subsequently imported into the country. However, encounters with exotic diseases in the UK were viewed as abnormal. Exotic diseases were contrasted with diseases horse owners could experience on a daily basis, with one participant noting they were *“something that your horse would not contract walking around a field”* (Leisure owner, ID 62).

Geographic boundaries were considered important when deciding when diseases could be considered exotic. Some participants felt there could be varying definitions of exotic diseases, depending on the perspective taken:

*“Since the presence or absence of different diseases vary from region to region and country to country, what may be an exotic disease to some is not to others.”* (Competition owner, ID 270)

Exotic diseases were described as “foreign” and “alien” when considered from a UK-based perspective. Some participants thought they originated in Africa or the Far East, while others simply noted that they existed in regions beyond their own. However, there were conflicting ideas about how participants defined their own region, with some defining it as the UK constituent country where they lived (e.g. England or Scotland) while others considered it to include all of Europe.

While participants expected exotic diseases to exist in certain geographic regions, they were also associated with tropical climates. It was felt that exotic diseases were less likely to be encountered in the UK because they were “*not local to the British climate”* (Competition professional, ID 13) and instead *“from a far-away country in the sun”* (Competition owner, ID 396). Perhaps unsurprisingly, the term ‘exotic disease’ prompted initial reactions to the term ‘exotic’ without further indicating its meaning; for example: *“A disease that is usually more prevalent in more exotic places”* (Leisure owner, ID 159).

#### Exotic diseases are dangerous

Exotic diseases were thought to be unlike any others previously encountered in the UK. They were described as *“not run of the mill”* (Leisure owner, ID 77), nasty, and harmful, with one participant articulating they were a *“danger to my animals”* (Leisure owner, ID 289). There was a concern about the susceptibility of the UK horse population because of a lack of prior exposure to exotic diseases: *“They may be more easily picked up and spread ‘round due to lack of resistance to them”* (Competition owner, ID 382). Furthermore, participants felt unable to protect their horses from exotic diseases due to an assumed lack of available vaccines. Exotic disease incursions were expected to have potentially serious outcomes, with one participant suggesting that the term implied that infections would be fatal if contracted.

Exotic diseases were described as something which, collectively, the horse industry did not know enough about. Participants were concerned about the implications of any outbreak of disease with which the horse industry had no prior experience. In particular, exotic diseases were considered new territory for the UK, and perhaps would come with unusual clinical signs and would require specialist treatment. Participants admitted their own lack of familiarity with exotic diseases, saying for example: *“I don’t know a lot about them”* (Leisure owner, ID 355), and *“It isn’t a term I am aware of”* (Leisure owner, ID 107).

### Attitudes towards the risk of exotic diseases

Eighty-four percent of participants (*n* = 356) provided a response about their attitudes towards being affected by an exotic disease. Four categories were identified among the responses: 1) responsible horse owners prevent disease, 2) horse owners need support to stop disease spread, 3) risk depends on proximity to the ‘risky’ horse, and 4) some risk is inevitable.

#### Responsible horse owners prevent disease

Participants felt that being a responsible horse owner reduced the chance of their horse being affected by an exotic disease. A responsible horse owner was described as someone who was aware of health hazards, was experienced in horse care, and took actions to protect their horse(s) from infection. Willingness to follow and implement advice from others was seen as a hallmark of good horse-owning practice: *“I would listen to directives from government, affiliated organisations and vets and act on them to protect my horses”* (Competition owner, ID 280). Participants felt accountable for maintaining the health of their horse(s) and were ready to take action to keep their horse(s) healthy: *“Anything with obvious symptoms, I would spot quickly and get treated”* (Non-racing owner, ID 257).

An important area of responsibility for participants was maintaining good hygiene and biosecurity practices. Good practices included using their own equipment, cleaning anything in use by others, and preventing contact with other horses:

*“I am very conscious of biohazards and the fact that I do not allow other horses onto my property. I am careful not to let my horses mix with horses outside their own herd and neither do I give lifts to other horses in my horse box [horse transport].”* (Competition owner, ID 123)

While some participants expressed that they alone were responsible for the care of their horse(s) and could therefore implement their preferred biosecurity practices, others relied on a yard owner/manager to provide that care. The reliance on others to implement biosecurity practices sometimes conflicted with participants’ ability to be responsible:

*“Although I personally am strict re: biosecurity, I don’t trust other liveries are as stringent or think it’s a ‘big deal’ like I do. Also, past livery yard owners have been very lax and seemed to have little understanding as to why biosecurity is so important an issue.”* (Leisure owner, ID 261)

Participants were most confident when certain practices, such as isolation, were mandatory on their yard because it meant that measures were being taken to protect their horse(s). Compliance with yard protocol on biosecurity practices increased the confidence that they were effective:

*“New horses, or travelling horses, are put into isolation on our yard. So the chances of infection spreading is limited. In the twelve years I have been on the yard, we have never had an outbreak of any illness!”* (Competition owner, ID 369)

A particular concern was how the actions of other horse owners could contribute to their own risk of exotic diseases. It was felt that the spread of disease was due in part to ‘uneducated’ or ‘ignorant’ horse owners. Relying on other horse owners to be responsible left some feeling unprotected:

*“I feel vulnerable to other horse owners taking precautions and responsibility to prevent the spread of disease. Some owners have zero awareness of how easily disease can spread.”* (Leisure owner, ID 166)

Participants also expressed concerns about determining which horse owners were behaving responsibly. Without a clear idea of who could be trusted as a responsible horse owner, participants felt that their risk of being affected by exotic diseases was heightened:

*“Too many horse owners take no responsibility for the health of their animals or their movement even if they have been near infected horses. No way of controlling which horses are which, and who owns them.”* (Competition owner, ID 192)

#### Horse owners need support to stop disease spread

While acknowledging that each person was accountable for their own horse’s health, additional support from the horse industry was deemed important to optimise disease prevention. Participants commented on how the actions of equine stakeholders, such as veterinary surgeons and the government, could affect their horse’s risk of exotic diseases. Having confidence that stakeholders were ready and willing to undertake disease prevention efforts contributed to feeling less at risk. In particular, participants appreciated the support provided by their veterinary surgeon and felt confident in their knowledge: *“[I’m in an] area where educated vets are proactive in providing advice and education to horse owners”* (Leisure owner, ID 207). In the event of an outbreak, veterinary surgeons were viewed as trusted sources of advice:

*“We receive Facebook updates from [our veterinary practice] regularly and I am confident they would also alert their clients of risk and provide preventative measures.”* (Competition owner, ID 313)

Having access to a supportive and proactive veterinary community contributed to confidence in implementing disease prevention measures, which in turn led to a sense of being protected against exotic disease threats. In contrast, participants felt a lack of support from the UK government in implementing disease prevention and control measures. Given the differences between horses and other livestock, the government’s involvement in an equine exotic disease outbreak was viewed as unlikely. There were also conflicting views on the actual effectiveness of measures implemented by the government. Some participants were confident in disease prevention and control measures implemented at the border:

*“We are so far very lucky that due to strict equine movements abroad, monitoring and having high procedures in vaccinations, laboratory tests and movement controls, inspections of equines coming back into the UK, etc, that hopefully we can [continue to] prevent non-UK diseases from entering the UK.”* (Leisure owner, ID 237)

Others felt that while the procedures were in place, they were not actually being followed. In particular, the horse passport system was considered ineffective:

*“Because government is slack in the control of equines being transported into England from Ireland & Scotland from Europe, lack of trading standards checking passports, equines being bought and sold from dealers without passports – no traceable history.”* (Leisure owner, ID 155)

Lastly, uncertainty about the effects of Brexit [withdrawal of the UK from the European Union (EU)] on horses travelling internationally influenced the perceived effectiveness of controls implemented by the government. Some participants felt that leaving the EU would remove the barriers currently preventing the incursion of disease into the country:*“Brexit may mean no cross-border agreements or controls, so no barriers to spread!”* (Leisure owner, ID 234). Others felt that Brexit would allow the implementation of better disease control at the border: *“Because we are leaving the EU we can put in place stronger rules when we allow foreign horses into the country”* (Competition professional, ID 273).

#### Risk depends on proximity to the ‘risky’ horse

The ‘risky’ horse was posed as any horse carrying some type of perceived risk to participants’ own horses. Horses that travelled (nationally or internationally), participated in competitions, or were simply unfamiliar to the participant were considered ‘risky’. Efforts to maintain distance from risky horses included preventing horses from mixing with others: *“I would hope the fact that my horses live out on my own land with no other horses would go some way to reducing the risk of an exotic disease”* (Leisure owner, ID 329). Furthermore, horses kept in rural areas were seen as isolated from risk compared to those near other (and potentially riskier) horses:

*“My county of Essex has a lot of dealers and ports so horses come in from the continent regularly not always in good condition and I always try to keep alert and manage smart.”* (Competition professional, ID 303)

Leisure horse owners viewed their horses as separate from, and therefore less likely to be affected by an exotic disease than, horses participating in racing or competition activities:

*“I tend not to worry too much as the horses at my yard are privately owned leisure horses who travel within the UK and generally have fairly limited contact with others [….] But obviously there are always potential risks. New diseases are coming to light and being carried but ‘exotic’ illnesses I feel aren’t likely to travel to the small pleasure horse owner as readily.”* (Leisure owner, ID 82)

Leisure horse owners often felt less at risk of exotic diseases because they did not participate in equestrian competitions. In contrast, those who participated in competitions were concerned about the inherent risk associated with these types of activities:

*“My horses compete a lot so come into contact with many other horses, whose movements and history I am not aware of. Compared to pleasure horses that don't leave the yard/field I think they are therefore more likely to come into contact with all kinds of diseases. Though obviously I take steps to reduce that risk!”* (Competition owner, ID 25)

Participants felt that any horses that were simply unfamiliar to them posed a significant risk to their own horse’s health. The term ‘strange’ was often used to describe horses with an unknown history. As such, participants were wary of their horse(s) meeting strange horses because they could not be certain of the associated risk: *“I do not have any strange horses visiting or staying at my premises”* (Leisure owner, ID 236). In contrast, familiar horses were viewed as low risk, regardless of their actions. For example, repeated exposure to the same horses at local events or outings was associated with a lower risk than contact with strange horses: *“Although we compete, we are at local, low key events where we know most people and generally know horses’ backgrounds”* (Leisure owner, ID 354). However, one participant did find themselves questioning the logic of this:

*“I travel to local events where local/familiar people repeatedly compete. Begs the question though… how do I know where their horses have come from?”* (Competition owner, ID 211)

#### Some risk is inevitable

Despite all actions that could be taken to avoid exotic diseases, participants acknowledged that they came with an aspect of inevitability. Some felt that an exotic disease outbreak was unavoidable, even if they implemented disease prevention measures: *“My care standards are high, but if it’s going to happen it will”* (Competition owner, ID 393). Others felt that they could not protect their horse(s) from all risks, especially when they left their premises: *“When we go out it’s not always possible to prevent other people or horses from touching yours”* (Competition owner, ID 206). Thus, it was viewed as ‘bad luck’ to be affected by an exotic disease despite implementing biosecurity practices. The inability to completely protect their horse(s) from exotic diseases sometimes resulted in a sense of helplessness: *“You can do everything in your power to prevent something happening, this does not mean it will not happen”* (Non-racing owner, ID 250).

An aspect of particular concern for horse owners was the impact of climate change on the spread of vector-borne exotic pathogens. These concerns depended on where participants were located, with those in the south feeling at higher risk: *“We do live in the warm south-east though and I think West Nile virus can be carried by birds and there have been some cases in Europe”* (Leisure owner, ID 74). Vectors, such as flies and mosquitoes, were seen as one of the biggest risks: *“Even with good practices in place, there is always some risk of exotic diseases, especially those spread by insect vectors*” (Competition owner, ID 298). Some felt it was impossible to completely isolate their horse from risk because of the difficulty in controlling large numbers of insects: *“Depends where the outbreak occurs as some of these diseases are spread by vectors which are very difficult to prevent biting another horse”* (Leisure owner, ID 254).

## Discussion

Our findings suggested that horse owners in this study perceived exotic diseases as an unfamiliar and dangerous threat, but one that was less likely to directly affect them given their existence was based elsewhere. Participating horse owners considered preventive measures were a hallmark of responsible horse-owning behaviour. However, they also worried about how factors beyond their control (e.g. actions of others, proximity to risky horses, and inevitability of an incursion) affected their risk of exotic diseases. Thus, exotic diseases were framed as something which could not be prevented solely by the actions of individual horse owners. These findings suggest several areas that could be targeted for risk communication and improved disease preparedness.

Participants’ perceptions of exotic diseases often included initial reactions to the term ‘exotic’ on its own, prompting them to feel less likely to be affected because they were inherently foreign diseases. The terms used to describe health hazards can affect both perceptions of risk and the preventive measures used against this risk, as was the case during the influenza pandemic in 2009 [21, 22]. The initial use of the term ‘swine flu’ to describe the pandemic contributed to misconceptions that avoiding pigs or pork products could protect against exposure to infection [21, 23]. Therefore, communication strategies regarding exotic diseases should consider how the public might interpret the messages. Compared to experts (e.g. scientists), the public’s perception of risk is more often influenced by characteristics of the health hazard rather than technical estimates of the hazard occurring [19]. Risks that are perceived as unfamiliar and threatening are more likely to be seen as requiring political intervention because they are unmanageable at an individual level [19]. This has important implications for disease preparedness if the industry expects horse owners to take actions to prevent exotic diseases, but horse owners feel that the responsibility lies with regulatory bodies.

Attitudes towards the role of the wider horse industry (e.g. veterinary surgeons and the government) varied depending on the level of trust and confidence in their actions. Some horse owners described a discrepancy between regulations implemented by the government and the actual efficacy of these protocols, with some feeling that the government would not assist in the event of an equine disease outbreak. The uncertain political climate at the time of the questionnaire (i.e. Brexit negotiations) may have increased the negative views towards the government’s involvement. Negative attitudes towards the government have also been demonstrated in other livestock industries, with some farmers attributing blame for past epidemics [24] and others feeling sceptical about the advice they provided [25]. Despite the lack of confidence in the government, participating horse owners felt supported by their veterinary surgeon and named them as trusted sources of advice about disease prevention. This suggests that in the event of an exotic disease incursion, horse owners may be more inclined to follow directives from their veterinary practice compared to the government. A positive relationship between horse owners and their veterinary surgeon would also benefit those who felt they lacked knowledge of exotic diseases, since their veterinary surgeon could act as a resource.

An important component of reducing the risk of exotic diseases was practising disease prevention measures, and thus, having good disease management practices was seen as part of the identity of a ‘responsible’ horse owner. This finding parallels the beliefs of farmers in other livestock industries who felt that routine undertaking of disease prevention measures was considered good practice [24, 26, 27]. Consequently, practising disease prevention distinguished whether a producer was ‘good’ or ‘bad’ [24, 26]. In this study, the separation between ‘responsible’ and ‘irresponsible’ horse owners led to feelings of vulnerability because good disease prevention habits could be undermined by the actions of others. This was also seen in some horse owners’ need to distance their horse from horses they perceived as ‘risky’ to ensure their horse could not catch any pathogens. The lack of trust in others to uphold disease prevention suggests that efforts to improve disease preparedness may benefit from taking an approach to encourage collective action [24]. Strengthening communication across the industry can facilitate the sense of a collective identity, which could motivate some horse owners to adopt recommended biosecurity practices [28]. Thus, efforts to facilitate communication and community-building across the horse industry could be beneficial for improving disease preparedness.

Despite undertaking disease prevention measures, the perceived uncontrollable nature of exotic diseases led some horse owners to feel an incursion was inevitable. This type of fatalistic attitude has been demonstrated with other disease threats, including Hendra virus [14], equine influenza [29], and bovine tuberculosis [30]. Balancing the effort of implementing disease prevention measures, and the expected efficacy of those measures, can influence the choice to implement recommended practices for disease management [14, 27, 31]. However, some horse owners felt that particular aspects of exotic diseases could not be managed by individual horse owners alone (e.g. climate change). Given the perceived inevitable nature of exotic diseases, horse owners’ motivations to implement disease prevention specifically for exotic diseases may differ from those used to prevent endemic diseases. As such, education efforts should focus on providing horse owners with timely communications about when they might need to implement exotic-disease specific measures, and practical suggestions for how they should do so.

The conclusions of this study are drawn from the responses of two open-ended questionnaire questions, which prevented horse owners from clarifying, expanding, or discussing their responses. Because of this limitation, the findings should be interpreted as a first insight into the participating horse owners’ perceptions of, and attitudes towards, exotic diseases. Given that the findings are based on a sample of horse owners, they may not reflect the opinions of all horse owners in the UK. Previous research that used data extracted from the National Equine Database (which ended in 2012) described the UK horse owning population as predominately female and located in England [7, 8], which aligns with the characteristics of participants in our sample. However, other demographics (e.g. education and experience with horses) cannot be compared due to the limited information on the UK horse owning population. Given that our sample mainly represents highly educated and experienced horse owners, the results are unlikely to be generalisable to those who may have less education or are less experienced with horses. The potential for non-response bias could also mean that responses from horse owners that did not practise disease prevention measures might not be included. The tendency of participants to highlight their good disease prevention habits and attribute blame to others might suggest an overestimation of their own behaviours due to social desirability bias [32]. Thus, further qualitative investigations are required to gain a better understanding of the relationship between horse owners’ attitudes towards exotic diseases and the adoption of recommended prevention strategies.

## Conclusions

This study explored horse owners’ perceptions of, and attitudes towards, exotic diseases and their associated risks. While participating horse owners felt there was a low risk of being affected by an exotic disease, they were concerned about the dangers posed to their horse’s health. Undertaking disease prevention measures was viewed as an important component of responsible horse-owning behaviour. However, many horse owners felt that exotic diseases could not be prevented by the actions of individual horse owners alone. Therefore, improved communication among horse owners and stakeholders in the industry may assist in clarifying expectations for exotic disease-specific prevention measures. A collaborative approach to disease prevention among horse owners and stakeholders is recommended to improve disease preparedness within the industry.

## Methods

### Study population

Participants in this study were respondents to an online questionnaire regarding horse owners’ awareness and perceived risk of exotic diseases, which took place between April and July 2018 [33]. Individuals who owned or cared for horses, ponies, or donkeys (herein referred to as ‘horses’) were eligible to participate in the study if they were 18 years of age or older, lived in the UK at the time of the questionnaire, and did not participate in horse racing. Due to the absence of a sampling frame of UK horse owners, participation was dependent on viewing the advertised link to the study during recruitment. Several methods were used to recruit potential participants, including advertising a link to the study during equestrian events, distribution through equestrian media and online forums, and online promotion by equestrian charities and organisations. The study protocol was reviewed and approved by the Royal Veterinary College Social Sciences Research Ethical Review Board (URN SR2017–1528).

### Data collection

Both the questionnaire used in this study, and a detailed description of the quantitative analysis conducted on the closed-ended questionnaire questions, are published elsewhere [33]. Data relevant to this study were collected using two open-ended questions on the questionnaire: 1) “What does the term ‘exotic disease’ mean to you?” and 2) “How do you think your horse’s chance of getting an exotic disease within the next 5 years compares to the ‘average’ horse, and for which reasons?”. Participants must have responded to at least one of the open-ended questions in order to be included in the content analysis.

To ensure that participants responded with their own interpretation of the term ‘exotic disease’, participants answered the first question before being provided with a definition of the term. Once they provided their own interpretation, participants progressed to the next page of the questionnaire where the authors’ definition of exotic diseases (diseases which are not normally found in the UK) was provided. The strategic placement of the definition between the two questions ensured that participants’ interpretation of exotic diseases was based on their own views, but their risk judgement was based on a shared understanding of what was meant by the term ‘exotic disease’. Participants were unable to return to previous questions in the questionnaire after progressing to the next page, and therefore could not return to the first question after seeing the authors’ definition of ‘exotic disease’.

### Data analysis

At the completion of the study period, all responses eligible for inclusion in the study were imported into R version 3.5.1 [34]. Statistically significant differences (*p* value < 0.05) between demographic characteristics of included participants and those who were excluded on the basis of non-response were assessed using the Wilcoxon rank-sum test for continuous variables and the Chi-square test (or Fisher’s exact test, where appropriate) for categorical variables. Descriptive characteristics of the participants were summarised using frequency distributions for categorical variables and median values and interquartile ranges (IQR) for continuous variables.

Participants’ responses to the open-ended questions were imported into NVivo version 12.2.0 (QSR International Pty Ltd.) for data management. Data were analysed descriptively using qualitative content analysis, which is an approach used to identify trends and patterns within textual data [35, 36]. Through the process of qualitative content analysis, data are classified into categories that represent similar meanings [37]. Thus, the analysis focused on describing the sentiments expressed by participants rather than quantifying how frequently they were mentioned. First, the responses were read several times to become familiar with the data. Data were coded in an inductive manner by assigning key words or phrases to describe the topic(s) mentioned by participants in their responses. All codes were reviewed and subsequently grouped into higher-order categories by taking an interpretive approach to identify patterns among the data [35, 37]. The categories and their associated codes were reviewed to ensure that the chosen structure accurately represented the data. The first author initially analysed the data and held discussions with the research team to finalise the categories. Quotations from the raw data are used throughout the text to illustrate concepts within each category. Omissions or insertions from the first author are included in square brackets to increase clarity of the statements.

## Data Availability

The data are not publicly available as they contain confidential participant information. However, the data are available from the corresponding author on reasonable request.

## Abbreviations

AHS: African horse sickness
EIA: Equine infectious anaemia
EU: European Union
IQR: Interquartile range
UK: United Kingdom
WNV: West Nile virus
